# Physiological, psychological and social negative effects of passive aging

**DOI:** 10.1192/j.eurpsy.2026.12220

**Published:** 2026-07-31

**Authors:** A. M. Konovalova, S. D. Tomilovskiy, V. D. Vinogradova

**Affiliations:** 1 Faculty of Psychology, Lomonosov Moscow State University; 2 Department of Pedagogy and Medical Psychology, Sechenov First Moscow State Medical University; 3 Department of Pedagogy and Medical Psychology, I.M.Sechenov First Moscow State Medical University of the Ministry of Health of the Russian Federation (Sechenovskiy University), Moscow, Russian Federation

## Abstract

**Introduction:**

Aging as a stage of the life cycle can proceed differently depending on the individual, psychological and social characteristics of a person. In recent decades increasing attention has been paid to the types of behavioral adaptation in old age, in particular to the difference between active and passive aging. The sociodemographic situation in the world, along with the development of the health-saving technologies, makes successful aging not only possible, but also necessary for the elderly with a high degree of their involvement in the public life.

**Objectives:**

To identify negative consequences of passive aging both for the elderly person and for society.

**Methods:**

The primary method employed is bibliographic analysis.

**Results:**

Active aging implies maintaining activity in different spheres of life, including physical, social, cognitive, and emotional domains, and is a well-studied construct.

In contrast, passive aging has been studied less. Research shows that it is accompanied with decreased activity, social isolation and closeness.

Passive aging often leads to emotional difficulties: increased anxiety, depression, and feeling of loneliness. Passive aging can be caused by external circumstances (illness, loss of loved ones, unfavourable conditions) or internal factors, such as negative image of aging, low self-esteem, and attitude towards inevitable deterioration.

It is important to emphasize that the boundary between active and passive aging is not always rigid. The same person can exhibit different types of behavior depending on the situation, level of support, and personal resources.

**Conclusions:**

There can be identified the following consequences of passive aging:

- physiological (deterioration of health due to physical inactivity, increased risk of disease development, decreased life expectancy).
- psychological (loss of meaning in life, lack of self-importance, disappointment in how life has been lived, decrease of cognitive processes).
- social (a person’s separation from society, a decline in social activity, loss of significant social status).

**Disclosure of Interest:**

None Declared

